# Neuroprotective effects of bis(7)-tacrine against glutamate-induced retinal ganglion cells damage

**DOI:** 10.1186/1471-2202-11-31

**Published:** 2010-03-03

**Authors:** Jia Hua Fang, Xing Hua Wang, Zhi Rong Xu, Fa Gang Jiang

**Affiliations:** 1 Department of Ophthalmology, Union Hospital, Tongji Medical College, Huazhong University of Science and Technology, Wuhan 430022, China; 2 Department of Ophthalmology, The First People's Hospital of Jingzhou, Yangtze University, Jingzhou 434000, China

## Abstract

**Background:**

Glutamate-mediated excitotoxicity, primarily through N-methyl-D-aspartate (NMDA) receptors, may be an important cause of retinal ganglion cells (RGCs) death in glaucoma and several other retinal diseases. Bis(7)-tacrine is a noncompetitive NMDA receptors antagonist that can prevent glutamate-induced hippocampal neurons damage. We tested the effects of bis(7)-tacrine against glutamate-induced rat RGCs damage in vitro and in vivo.

**Results:**

In cultured neonatal rats RGCs, the MTT assay showed that glutamate induced a concentration- and time-dependent toxicity. Bis(7)-tacrine and memantine prevented glutamate-induced cell death in a concentration-dependent manner with IC50 values of 0.028 μM and 0.834 μM, respectively. The anti-apoptosis effects of bis(7)-tacrine were confirmed by annexin V-FITC/PI staining. In vivo, TUNEL analysis and retrograde labeling analysis found that pretreatment with bis(7)-tacrine(0.2 mg/kg) induced a significant neuroprotective effect against glutamate-induced RGCs damage.

**Conclusions:**

Our results showed that bis(7)-tacrine had neuroprotective effects against glutamate-induced RGCs damage in vitro and in vivo, possibly through the drug's anti-NMDA receptor effects. These findings make bis(7)-tacrine potentially useful for treating a variety of ischemic or traumatic retinopathies inclusive of glaucoma.

## Background

Glutamate is a major excitatory neurotransmitter in the central nervous system, including the retina[1,2]. It is released by the presynaptic cells and acts on N-methyl-D-aspartate (NMDA), α-amino-3-hydroxy-5-methyl-4-isoxazolepropionic acid (AMPA), and kainite (KA) receptors [3]. If excessive amounts of glutamate are released or if glutamate clearance is insufficient, neuronal death will result in a process known as excitotoxicity. The glutamate receptor-mediated excitotoxicity has been associated to various diseases of the brain and eye, which include Alzheimer's disease[4], retinal ischemia[5,6] and glaucoma[7,8]. Although retinal ganglion cells (RGCs) express all of three receptor subtypes, the glutamate toxicity is primarily mediated by NMDA receptors [9-11].

Glaucoma, a neurodegenerative disease[12], is associated with selective death of retinal ganglion cells [13]. The disease is characterized by an elevation in intraocular pressure (IOP), which leads to increased glutamate levels [14]. Vitreal glutamate levels are elevated in dogs[15] and humans with primary glaucoma [16], and also in monkeys with experimentally induced chronic glaucoma[16]. Lowering IOP is the current main treatment for glaucoma, yet disease progression continues to occur even in patients with significant IOP reduction[17]. Therefore lowering IOP is inadequate for glaucoma patients [12,18]. Efforts have been made to attempt to discover appropriate drugs or chemicals (neuroprotectants) that can be taken orally to slow down retinal ganglion cell death and have negligible side-effects [19]. Memantine is an uncompetitive NMDA receptor antagonist which is prescribed for the treatment of Alzheimer's disease [20]. However, two recent parallel clinical trials conducted to test the efficacy of memantine as a neuroprotectant for glaucoma were unsuccessful[21]. The results of the trials showed that memantine had no significant effects in preserving visual function.

Until now, there has been no neuroprotective agent indicated for the treatment of glaucoma. A neuroprotectant that has a single mode of action like memantine has a limited positive effect in slowing down ganglion cell death[19]. Pharmacological agents that simultaneously affect multiple biological mechanisms are consequently desired. This has been referred to as the "cocktail" approach [22]. One-drug-multiple-targets approach in the treatment of neurodegenerative diseases is possible way forward [19,23].

Beside NMDA receptor antagonism, other strategies have been investigated in the development of neuroprotective therapies, which include voltage-dependent calcium channel blockade [24,25], nitric oxide synthase (NOS) inhibition [26,27], and so on. Bis(7)-tacrine (1,7-N-heptylene-bis-9,9'-amino-1,2,3,4-tetrahydroacridine), a dimeric acetylcholinesterase (AChE) inhibitor derived from anti-Alzheimer's drug tacrine, possesses remarkable neuroprotective activities through concurrent inhibition of AChE [28,29], NMDA receptor[30] and nitric oxide synthase [31,32]. Moreover, bis(7)-tacrine attenuates neuronal apoptosis by regulating L-type voltage-dependent calcium channels(VDCCs) [33]. Recent studies showed that bis(7)-tacrine prevented glutamate-induced excitotoxicity by selectively inhibiting NMDA receptors in primary cultured cerebellar granule neurons (CGNs), without the involvement of the other two ionotropic glutamate receptors, AMPA receptor and KA receptor [30,34,35].

Based on these evidence, we hypothesis that bis(7)-tacrine attenuate glutamate-induced retinal ganglion cells damage through the blockade of NMDA receptors. We tested the effect of bis(7)-tacrine in two models of glutamate excitotoxity, RGCs in culture with glutamate and intravitreal injection of glutamate. The results showed that bis(7)-tacrine reduced glutamate-induced retinal ganglion cells damage in vitro and in vivo.

## Results

### Identification of cultured RGCs

**Figure 1:**
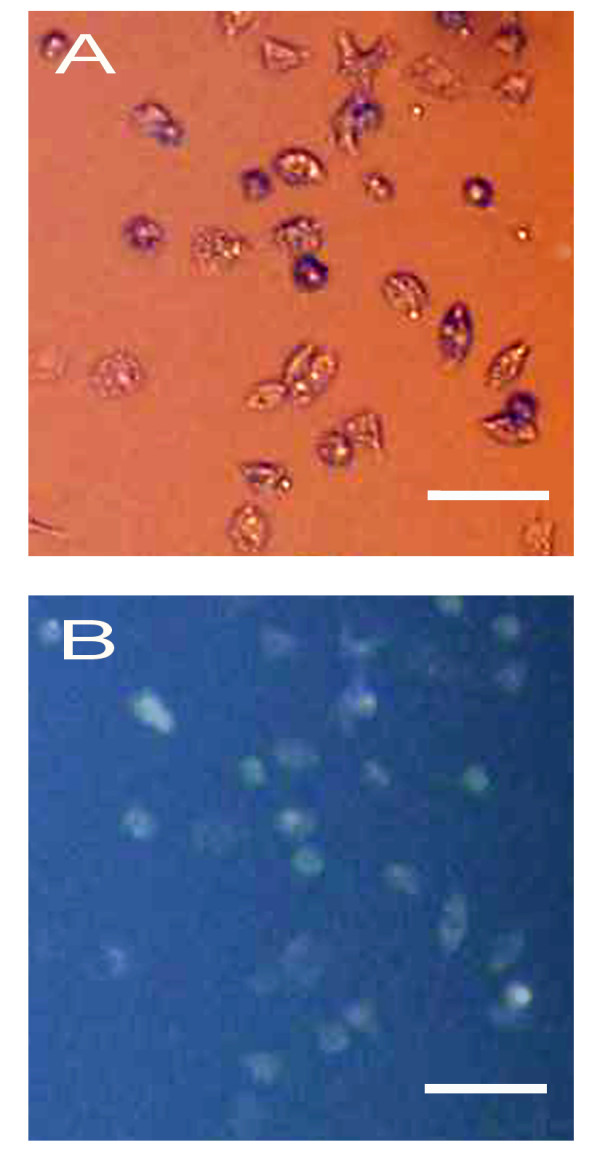
**Identification of cultured rat RGCs**. Phase contrast image (A) and fluorescence image (B) of RGCs purified from a 5-day-old rat that had received injections of fluorogold into the superior colliculi 1 day after birth. The cells were cultured for 1 days under control conditions. Scale bar, 50 μm.

The purity of isolated RGCs was assessed by fluorescent microscope (IX71, Olympus, Japan) using a UV filter that permitted the visualization of fluorogold fluorescence (Fig. 1). RGCs were labeled by fluorogold in a retrograde manner. After a two-step immunopanning procedure, approximately 87.8% (1896/2158 cells) of the collected cells were labeled by fluorogold. Because it is difficult to obtain uniform labeling, a small number of RGCs were probably not identified by retrograde transport, and the purity of the preparations was therefore underestimated.

### Bis(7)-tacrine prevents glutamate-induced cell death more potently than memantine

**Figure 2:**
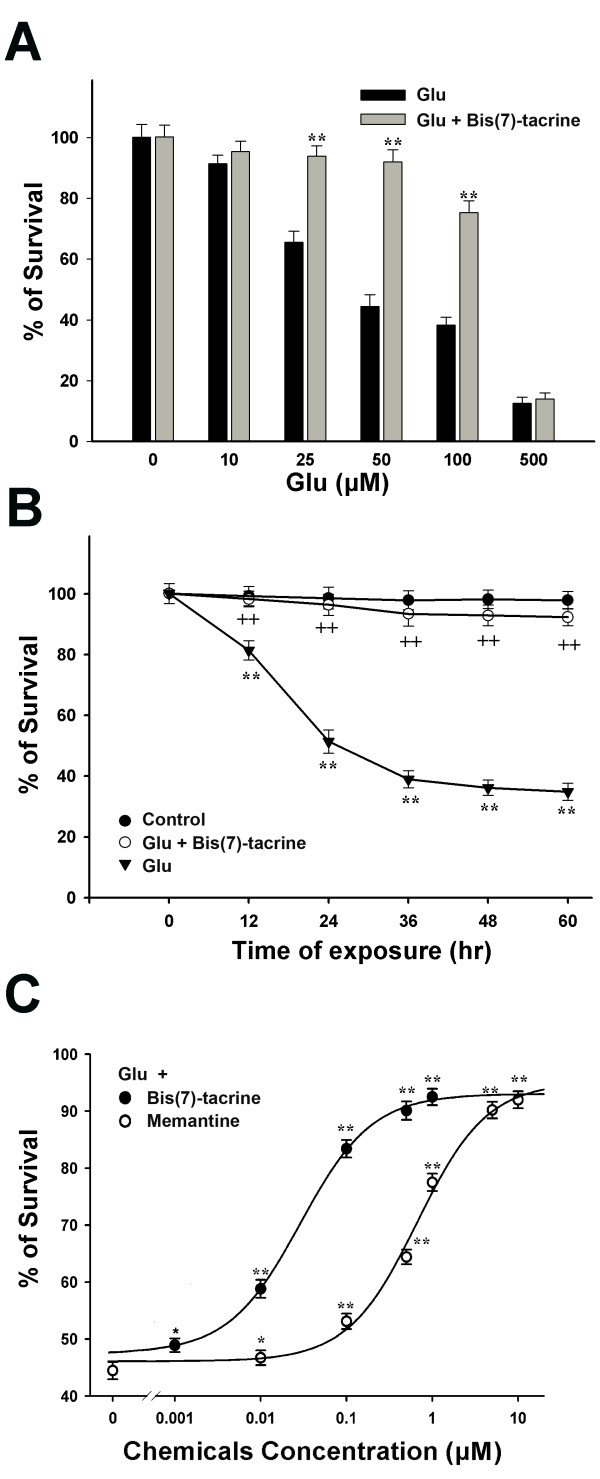
**Glutamate (Glu) induces a concentration- and time-dependent changes and bis(7)-tacrine is more potent than memantine in preventing the excitotoxicity of glutamate in the MTT assay**. A, at 3 days in vitro, cultured RGCs were exposed to 1 μM bis(7)-tacrine for 2 h before the addition of glutamate at the different concentrations indicated. Cell viability was measured at 24 h by MTT assay. B, compared with normal neurons, RGCs were preincubated with or without 1 μM bis(7)-tacrine for 2 h and then exposed to 50 μM glutamate at different times indicated. Cell viability was measured at the indicated times. C, graph plotting percentage of neuron survival as a function of concentrations of bis(7)-tacrine(●)and memantine(○). RGCs were pre-treated with bis(7)-tacrine (0.001-1 μM) or memantine (0.01-10 μM) for 2 h before the addition of 50 μM glutamate. At 24 h after the challenges, cell viability was measured. All of the data, expressed as percentages of the corresponding control, were means ± SEM of three separate experiments. *P < 0.05, **P < 0.01 versus glutamate group in A and C or versus control at the same time in B. ++ P < 0.01 versus glutamate group at the same time in B.

Glutamate toxicity was found to be concentration- and time-dependent, which was consistent with previous reports[9,36]. At 3 days in vitro, RGCs were exposed continuously to 25-300 μM glutamate for 24 h and 50 μM glutamate alone at different times. Cell viability was measured by MTT assay. Glutamate induced a concentration- dependent loss in neuronal viability starting from 25 μM and peaking at 300 μM (Fig. 2A). Glutamate at 50 μM induced a time-dependent neuronal death starting at 12 h and culminating at 36 h (Fig. 2B). RGCs were pretreated with 1 μM bis(7)-tacrine for 2 h and exposed to different concentrations of glutamate for 24 h. With the increase of glutamate concentrations, the neuroprotective effects of bis(7)-tacrine gradually decreased and completely lost when RGCs were exposed to over 300 μM glutamate (Fig. 2A). Bis(7)- tacrine at 1 μM retained its neuroprotective effects for at least 60 h after glutamate stimulation (Fig. 2B). RGCs were pretreated with the gradually increasing concentrations of bis(7)-tacrine or memantine for 2 h and then exposed to 50 μM glutamate for 24 h. It was found that bis(7)-tacrine and memantine prevented glutamate-induced cell death in a concentration-dependent manner with IC50 values of 0.028 μM and 0.834 μM, respectively (Fig. 2C). Bis(7)-tacrine inhibited the glutamate-induced cell death more potently than memantine.

### Bis(7)-tacrine reduces glutamate-induced apoptosis of RGCs in vitro

**Figure 3:**
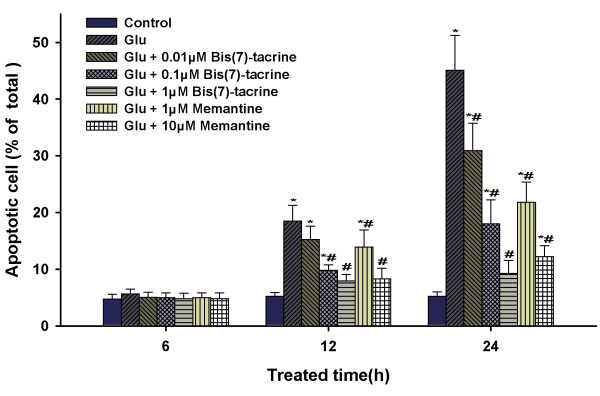
**Bis(7)-tacrine reduces glutamate(Glu)-induced apoptosis of RGCs in vitro**. RGCs were cultured with and without glutamate for 6-24 h in the presence or absence of bis(7)-tacrine (0.01-1 μM) or memantine (1-10 μM). Percentage of apopotic cells was detected by Annexin-V FITC/PI staining by flow cytometry. Apoptotic cells included Annexin V(+)/PI(-) and Annexin V(+)/PI(+) cells. Results were expressed as the means ± SEM of 7 independent experiments. (*P < 0.05 as compared to the control group for each time point; # P < 0.05 as compared to the glutamate group for each time point).

The anti-apoptotic effects of bis(7)-tacrine were studied using an Annexin-V FITC/PI assay. The results demonstrated that 50 μM glutamate induced apoptosis in a time-dependent manner and bis(7)-tacrine could prevent glutamate-induced apoptosis (Fig. 3). Although the initial 6 h of glutamate exposure did not change the percentage of apoptotic cells relative to the control group, there were marked increases in the percentage of apoptotic cells after 12 h (18.48 ± 2.76%; p < 0.05) and 24 h (45.12 ± 6.15%; p < 0.05). Bis(7)-tacrine inhibited glutamate-induced damage to RGCs in a concentration-dependent manner. At 12 h, bis(7)-tacrine at 0.01 μM has a tendency to reduce glutamate-induced damage(P > 0.05), but bis(7)-tacrine (0.1 μM, 1 μM), as well as memantine (1 μM,10 μM), decreased the percentage of apoptotic cells to 9.78 ± 0.97%, 7.92 ± 1.14%, 13.92 ± 2.98%, 8.28 ± 1.87% respectively (all p < 0.05). At 24 h, all the concentrations of two drugs, bis(7)-tacrine (0.01 μM, 0.1 μM, 1 μM) and memantine (1 μM,10 μM), reduced effectively the percentage of apoptotic cells, but only with bis(7)-tacrine (1 μM) show no significant increase in the percentage of apoptotic cells in comparison with control group.

### Bis(7)-tacrine reduces glutamate-induced apoptosis of RGCs in vivo

**Figure 4:**
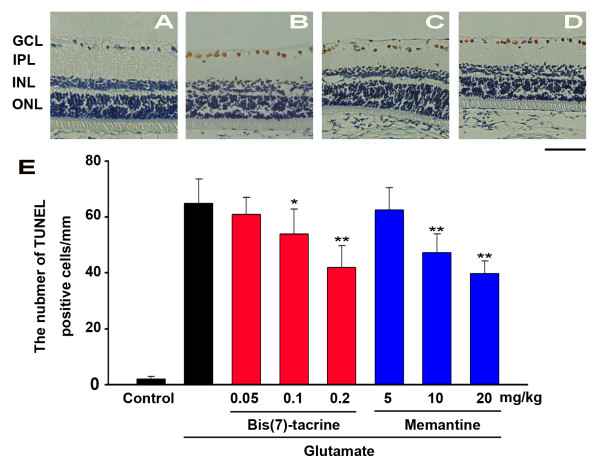
**Bis(7)-tacrine reduces glutamate(Glu)-induced apoptosis of RGCs in vivo**. Eyes were enucleated at 18 h after intravitreal injection of glutamate(Glu) (20 nmol) or vehicle. TUNEL staining was performed and TUNEL-positive cells were counted in GCL. (A) A representative photograph of retina in the vehicle group. (B) A representative photograph of retina in the glutamate group. (C & D) A representative photograph of retina in the glutamate group with peritoneal injection of bis(7)-tacrine(0.2 mg/kg)(C) or memantine(20 mg/kg)(D). (E) The number of TUNEL-positive cells in GCL was counted. Each column represents means ± SEM (n = 6). *P < 0.05, **P < 0.01 versus glutamate group alone. GCL: ganglion cell layer, IPL: inner plexiform layer, INL: inner nuclear layer, ONL: outer nuclear layer. Scale bar, 50 μm.

Apoptotic changes in the retina were assessed by the TUNEL method. Little TUNEL positivity was observed in the control (PBS-injected) group (Fig. 4A). However, at 18 h after glutamate was injected into the eyes, prominent TUNEL-positive cells were found in the ganglion cell layer(GCL) and the inner nuclear layer (INL) (Fig. 4B). The density of TUNEL-positive cells in GCL was 64.87 ± 8.83 cells/mm in the glutamate group whereas it was 54.50 ± 8.15/mm in the animals receiving bis(7)-tacrine at 0.1 mg/kg (P <0.05) and 42.87 ± 7.82/0.5 mm when the dose of bis(7)-tacrine was 0.2 mg/kg (P < 0.01). Compared with the glutamate group, there was no statistically significant reduction when animals receiving bis(7)-tacrine at 0.05 mg/kg (P > 0.05). The results showed that bis(7)-tacrine rescued these neurons from apoptosis which was induced by glutamate in a dose-dependent manner. Memantine (10 mg/kg, 20 mg/kg) showed the same protective effects as bis(7)-tacrine(0.2 mg/kg) (Fig. 4E).

### Bis(7)-tacrine protect RGCs in experiments using retrograde labeling of RGCs

**Figure 5:**
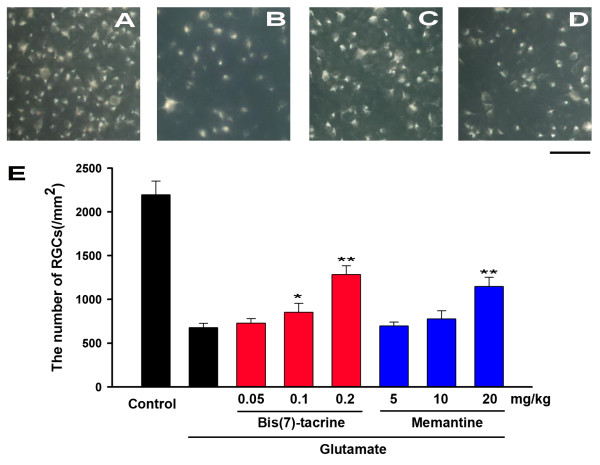
**Effect of bis(7)-tacrine on RGCs following intravitreal injection of glutamate(Glu) (20 nmol)**. (A) A representative photograph of RGCs labeled with fluorogold injected into the superior colliculus in normal retina. (B) A representative photograph of fluorogold -labeled RGCs 7 days after intravitreal injection of glutamate. (C & D) A representative photograph of fluorogold -labeled RGCs 7 days after intravitreal injection of glutamate with daily peritoneal injection of bis(7)-tacrine(0.2 mg/kg)(C) or memantine(20 mg/kg)(D). (E) The number of RGCs was counted. Each column represents means ± SEM (n = 6). *P < 0.05, **P < 0.01 versus glutamate group alone. Scale bar, 50 μm.

The GCL is composed of about equal numbers of RGCs and displaced amacrines[37]. To ensure accurate identification of RGCs, we retrogradely labeled the RGCs in rats with fluorogold allowing us to selectively label greater than 99% of the RGCs. In PBS-injected eyes without glutamate, the mean density of RGCs was 2196.6 ± 155.0 cells/mm^2 ^(n = 6) (Fig. 5A). Fifteen minutes after intraperitoneally injection of bis(7)-tacrine(0.05, 0.1, 0.2 mg/kg), memantine(5, 10, 20 mg/kg) or PBS, 20 nmol glutamate was injected intravitreally into the same animals. At 7 days after glutamate injection, the mean densities of the RGCs in control (PBS-injected) and experimental (0.05, 0.1, 0.2 mg/kg bis(7)-tacrine and 5, 10, 20 mg/kg memantine) eyes were 676.5 ± 49.6, 729.3 ± 50.5, 853.0 ± 102.0, 1284.6 ± 99.0, 697.3 ± 42.4, 770.0 ± 85.2, 1148.2 ± 105.0 cells/mm^2^(n = 6) (Fig. 5E), respectively, and the different was statistically significant (PBS vs 0.1 mg/kg bis(7)-tacrine, P = 0.038; PBS vs 0.2 mg/kg bis(7)-tacrine, P < 0.01; PBS vs 20 mg/kg memantine, P < 0.01). These data showed that both of bis(7)-tacrine and memantine possessed neuroprotective effects against glutamate-induced RGCs death, and bis(7)-tacrine (0.2 mg/kg) had the same effects as memantine (20 mg/kg).

## Discussion

Glutamate excitotoxicity is thought to contribute to a broad variety of neurological diseases, including Alzheimer's disease and glaucoma. In retina tissues, the predominant form of glutamate neurotoxicity is mediated by overstimulation of the NMDA subtype of glutamate receptors, which in turn causes an increase of Ca^2+ ^influx, followed by cell death [5,9,38]. Recent research demonstrated that bis(7)-tacrine prevented glutamate-induced cerebellar granule neurons (CGNs) apoptosis through directly blocking NMDA receptors [30,34]. We show here that bis(7)-tacrine provided in vitro and in vivo neuroprotective effects on glutamate-induced RGCs damage. Although the cytoprotective actions of bis(7)-tacrine have previously been observed in brain neurons under excitotoxic/ischemic conditions[23,30,34,39], to our knowledge, this is the first report to elucidate the neuroprotective action of bis(7)-tacrine on RGCs in response to glutamate excitotoxicity.

Some researches have reported that RGCs are highly susceptible to glutamate toxicity in vitro and in vivo[9,38,40]. First of all, we explored the in vitro effectiveness of bis(7)-tacrine against glutamate-induced RGCs damage. In a previous report, glutamate toxicity was concentration-dependent with a calculated EC_50 _of 30.8 μM[9]. In our study, the MTT assays have shown that cultured RGCs are highly sensitive to 24 h 50 μM glutamate treatment, which produces 50% or greater cell death. Furthermore, bis(7)-tacrine, like the NMDA receptor antagonist memantine, has been confirmed to attenuate the cytotoxic effect of glutamate. As memantine showed affinity and potency comparable to those of bis(7)-tacrine in blocking the NMDA receptor [39], we chose memantine as the positive control in this study. The minimal effective concentration of bis(7)-tacrine against glutamate excitotoxicity was approximately 0.01 μM. The combination of data from the MTT assays and the Annexin V-FITC/PI assays, provided strong evidence that bis(7)-tacrine was significantly more potent than memantine in inhibiting glutamate-induced cell damage. This finding was similar to previous reports in cultured rat cortical neurons[34].

Next, we verified the neuroprotective effects of bis(7)-tacrine in animal models of glutamate-induced retinal injury. The pharmacokinetic research in the body of the rat showed that bis(7)-tacrine was rapidly and widely distributed to its target tissues such as brain[41,42]. In a model of acute focal cerebral ischemic insults in MCAO rats, bis(7)-tacrine at doses of 0.1 mg/kg and 0.2 mg/kg significantly reduced ischemic impairment in vivo[23]. In the current study, two animal assays showed that bis(7)-tacrine(0.2 mg/kg) protected RGCs from glutamate excitotoxicity, while bis(7)-tacrine(0.05 mg/kg) had no significant neuroprotective effects.

We cannot definitively state that bis(7)-tacrine was neuroprotective for retinal ganglion cells alone. In retina tissues, several cells express NMDA receptors, such as RGCs and displaced amacrine cells[43] in the retinal ganglion cell layer (GCL)and bipolar cells[44] in the inner nuclear layer (INL). These cells are susceptible to excitotoxic cell death, we did not differentiate between retinal ganglion cells and other cells.

Neuroprotection was initially studied as a treatment strategy for various neurological disorders including stroke, dementia, multiple sclerosis, Alzheimer's disease and glaucoma. Despite successful preclinical cell culture and animal model experiments, most of these therapies were not successful at the clinical stage of testing because of either unacceptable side effect profiles or a lack of efficacy [12,45,46]. High affinity NMDA receptor antagonists such as MK-801 are undesirable as they entail nonselective inhibition of tonic glutamate activity as well as phasic physiological NMDA receptor function [47]. Memantine is a moderate affinity, uncompetitive NMDA receptor antagonist prescribed for the treatment of moderate to severe Alzheimer's disease [48,49]. However, recent clinical findings showed that there was no clear benefit after glaucoma patients received memantine; thereby it was suggested that neuroprotectants with multiple modes of actions were likely to reveal clearer results than was found for memantine [19].

The preclinical studies demonstrated that bis(7)-tacrine had low toxicity in animal models[50], and bis(7)-tacrine possessed multiple physiological activities including anti-NMDA receptors, anti-AChE, anti-L-type-voltage-dependent calcium channels(VDCCs), anti-nitric oxide synthase (NOS) signaling and the regulation of the downstream signal of NMDA receptors [28,30,32,33,51]. In the present study, bis(7)-tacrine prevented glutamate-induced RGCs damage possibly by inhibiting NMDA receptors. At this point, electrophysiology studies will be needed to verify the blocking kinetics of bis(7)-tacrine on three glutamate receptors in the purified RGCs. What's more, further calcium imaging studies will be required to determine the role of calcium permeation through NMDA receptors and to determine if intracellular calcium is involved in triggering neuroprotection or inhibiting glutamate excitotoxicity. Besides inhibiting NMDA receptors, the other effects of bis(7)-tacrine on RGCs deserves further study.

## Conclusions

In conclusion, our experiments have demonstrated that bis(7)-tacrine can provide neuroprotection against glutamate-induced retinal ganglion cells damage in vitro and in vivo. The effects may be achieved through inhibitions of NMDA receptors. This neuroprotective effects of bis(7)-tacrine may lead to a novel approach for the treatment of retinopathies, such as glaucoma.

## Methods

### Animals and reagents

Sprague-Dawley(SD) rats, including 1-3 days rats and adult male rats, were obtained from the Animal Center in Tongji Medical College, Huazhong University of Science and Technology, and were housed in the animal facility under standard conditions of room temperature and a 12:12 h light-dark cycle with free access to food and water. All animal experiments followed the guidelines for the care and use of animals established by Tongji Medical College, Huazhong University of Science and Technology and adhered to the tenets of the Declaration of Helsinki. Bis(7)-tacrine was purchased from Cayman Chemical Co.(USA). Fluorogold was purchased from Biotium (Hayward, USA). Unless noted, all other reagents were obtained from Sigma (St. Louis, MO, USA).

### Cell culture and purification of RGCs

RGCs from retinas of 1-3 days rats were purified by a two-step immunopanning procedure. Briefly, the retinal tissue was dissociated into single cells in EMEM (Gibco, China) containing 15 U/ml papain and 70 U/ml collagenase. The dissociated cells were incubated in a polypropylene tube coated with an anti-rat macrophage monoclonal IgG (Chemicon International, Inc, CA, USA) to exclude macrophages, and then incubated in a tube coated with an anti-rat Thy 1.1 monoclonal IgG (Chemicon International, Inc, CA, USA). The tube was gently washed with PBS for five times, and adherent RGCs were collected by centrifugation at 600 g for 5 minutes.

Before the examinations of effects of bis(7)-tacrine on RGCs, a preliminary study was conducted to determine purity of RGCs after the two-step immunopanning procedure. After 1-day-old rats were anesthetized by intraperitoneal injection with 10% (w/v) chloral hydrate (350 mg/kg), RGCs were labeled in a retrograde manner by injecting 4% fluorogold into the superior colliculi. Four days later, after this immunopanning method, approximately 87.8% (1896/2158 cells) of the collected cells were labeled by fluorogold. Next, in further examinations of effects of bis(7)-tacrine on RGCs, RGCs were used from rats without fluorogold injection and grown in serum-free medium (Gibco-China), containing 1 mM glutamine, 10 μg/mL gentamicin, B27 supplement (1:50), 40 ng/mL each of BDNF and CNTF, and 5 μM forskolin. RGCs were cultured in a CO_2 _incubator (Thermo Lab 2300, USA) with 5% CO_2 _at 37°C. Before seeding, the plates were coated with poly-D-lysine (PDL, 70 kDa, 10 μg/ml) at room temperature followed by overnight incubation with mouse laminin. RGCs were cultured for 3 days and then exposed them to glutamate and/or bis(7)-tacrine for MTT assay or annexin-V FITC/PI assay.

### Measurement of neurotoxicity

The percentage of surviving RGCs in the presence of bis(7)-tacrine and/or glutamate was estimated by determining the activity of mitochondrial dehydrogenases with 3(4,5-dimethylthiazol-2-yl)-2.5-diphenyltetrazolium bromide (MTT) assay [30]. Cells were seeded at a density of 5000 cells/well in 96-well plates. The assay was performed according to the specifications of the manufacturer (MTT kit I; Roche China, Ltd.). Briefly, the rat RGCs were cultured in 96-well plates, 10 μl of 5 mg/ml MTT labeling reagent was added to each well containing cells in 100 μl of medium, and the plate was incubated for 4 h in a humidified incubator at 37°C. After the incubation, 100 μl of the solvating solution (0.01 N HCl in 10% SDS solution) was added to each well for 17-18 h. The absorbance of the samples was measured at a wavelength of 570 nm with 630 nm as a reference wavelength. Unless otherwise indicated, the extent of MTT conversion in cells exposed to glutamate is expressed as a percentage of the control.

### Annexin-V FITC/PI assay

Apoptosis was detected using an Annexin-V FITC/PI detection kit (Jiancheng Biotechnology Co., Ltd., Nanjing, China) according to the manufacturer's directions. The cells were digested with 0.125% trypsin, washed with ice-cold phosphate-buffered saline and resuspended in binding buffer (5 × 10^5 ^cells/ml). Then, the cells were centrifuged at 1,000 rpm for 5 min at 4°C. After the supernatant had been discarded, 500 μl of binding buffer, 5 μl of annexin-V-FITC and 5 μl of propidium iodide (PI) were added to 200 μl of the cell suspension. After mixing gently, the suspensions were incubated for 15 min at room temperature without light. Finally, the cells were analyzed by flow cytometry (BD LSRII; BD Biosciences).

### Intravitreal administration of glutamate

Male rats (220-280 g) were anesthetized by intraperitoneal injection of 10% (w/v) chloral hydrate (350 mg/kg) and rectal body temperature was maintained at 37°C with a heating pad during the experiments. The pupils were dilated with tropicamide and a single dose of 5 μl of 4 mM glutamate (total amount 20 nmol) in 0.01 M PBS (pH 7.4) was injected into the vitreous cavity using 32-gauge Hamilton needle and syringe. PBS was administered as a control.

### TUNEL staining

TUNEL staining was performed according to the manufacturer's protocols (In Situ Cell Death Detection Kit; Roche China, Ltd.) to detect retinal cell apoptosis induced by glutamate. Twenty-four rats were divided into eight groups: three bis(7)-tacrine-treated groups (0.05, 0.1, 0.2 mg/kg), three memantine-treated groups (5, 10, 20 mg/kg), a control group, and a glutamate group. Six eyes per experimental condition were used. In this study, 18 h after the glutamate injection, rats were killed with an overdose of chloral hydrate. The eyes were immediately enucleated and fixed in 4% paraformaldehyde in PBS for the TUNEL studies. The specimens were then dehydrated and embedded in paraffin and 5 μm sections were cut. These sections were stained by the TUNEL method according to the manufacturer's directions. The yellow condensed TUNEL positive cells were counted under a 20× objective microscope. No attempt was made to distinguish the cell types in the GCL, and displaced amacrine cells were not excluded from the counts. To minimize the variance in cell number, we counted TUNEL positive cells in the retinal ganglion cell layer (GCL) manually at 1.0-2.0 mm (both sides) from the center of the optic disc. The average number of TUNEL positive cells/eye was obtained from three sections of each retina.

### Retrograde labelling and counting of RGCs

To examine the change in the number of RGCs after glutamate injection, RGCs were retrogradely labeled with fluorogold. Twenty-four rats were treated respectively with the same division and administration above mentioned. Fifteen minutes before intravitreally injection of glutamate, drugs were intraperitoneally administered to the rats in a volume of 1.5 mL/kg body weight. Four days after the glutamate injection, retrograde labeling of RGCs was made. Briefly, rats were anesthetized by intraperitoneally injection with 10% (w/v) chloral hydrate (350 mg/kg) and then the heads were fixed in a stereotaxic apparatus. Fluorogold was microinjected bilaterally into the superior colliculi(SC) and dorsal lateral geniculate nuclei (dLGN) of rats. Three days after the Fluorogold injection (seven days after the glutamate injection), the animals were killed by an intraperitoneal overdose injection of chloral hydrate and the eyes were enucleated. Eyes were fixed with 4% paraformaldehyde for 1 h. Retinas were removed from the sclera, divided into four radial cuts and mounted on slides. Analysis for the number of fluorogold-labeled RGCs was carried out. Briefly, Tracer-labeled RGCs counting was performed in 12 areas of 0.072 mm^2 ^each (three areas per retinal quadrant) at 2/6, 3/6, and 5/6 of the retinal radius under a fluorescent microscope (Olympus IX71). Data from 12 areas from each eye were averaged.

### Statistical analysis

Data are expressed as mean ± SEM. Statistical significance was determined using the ANOVA method followed by Dunnett's test in the case of multiple comparisons. Single comparisons were performed by Student's t test. Statistical analysis of concentration-response data was performed using the nonlinear curve-fitting program ALLFIT, which uses an ANOVA procedure[34,51]. Values reported for concentration-response analysis are those obtained by fitting the data to the equation:

where *X *and *Y *are concentration and response, respectively, *E*_max _is the maximal response, EC_50 _is the concentration yielding 50% of maximal effect (EC_50 _for activation, IC_50 _for inhibition), and n is the slope factor.

## Authors' contributions

JHF participated in the design of the study, carried out the cell assays and animal experiments, performed the statistical analysis, and wrote the manuscript. XHW assisted in the animal experiments and helped to draft the manuscript. ZRX assisted in the cell assays. FGJ (corresponding author) designed the study and directed the research. All authors read and approved the final manuscript.
